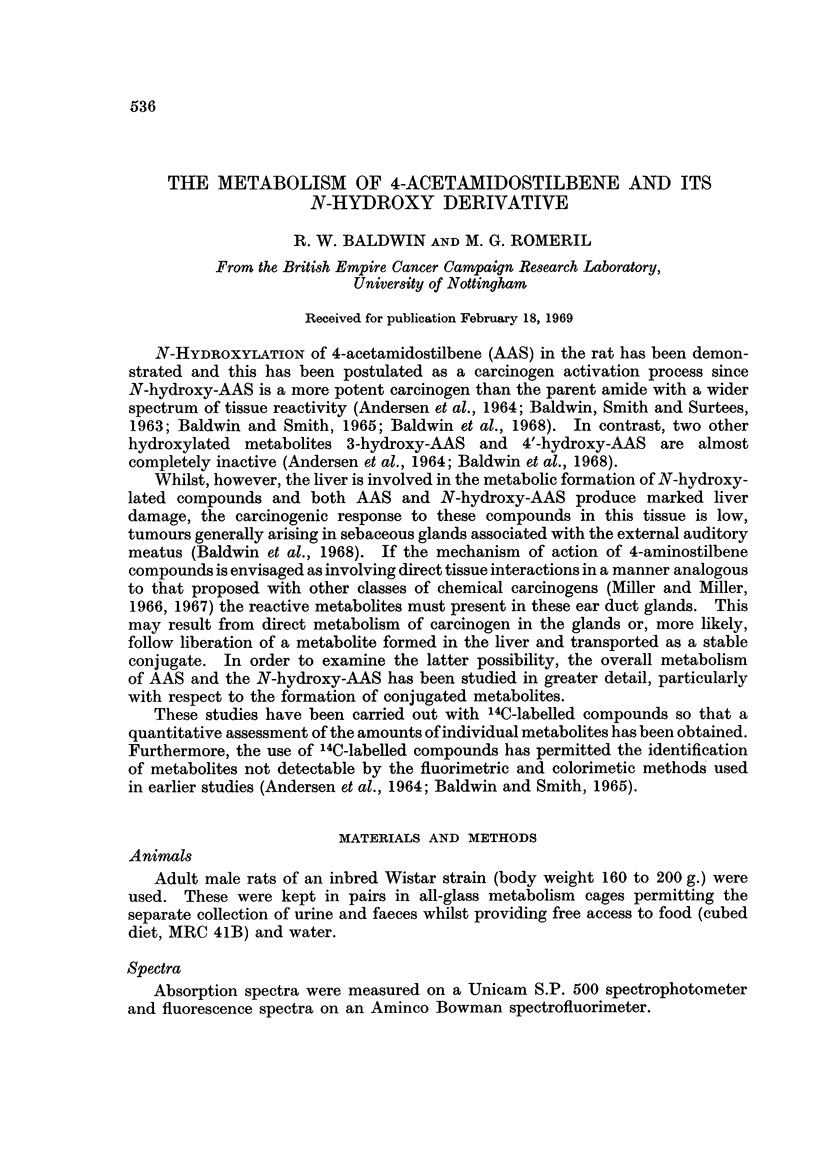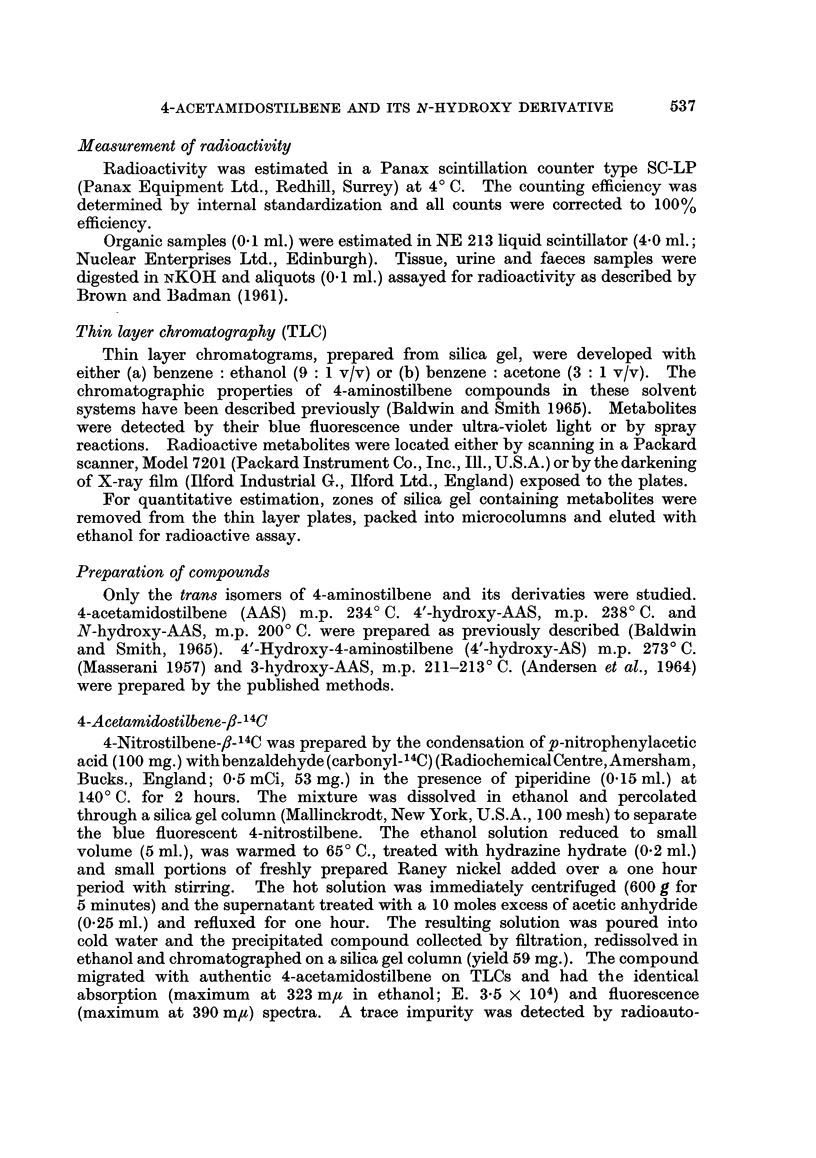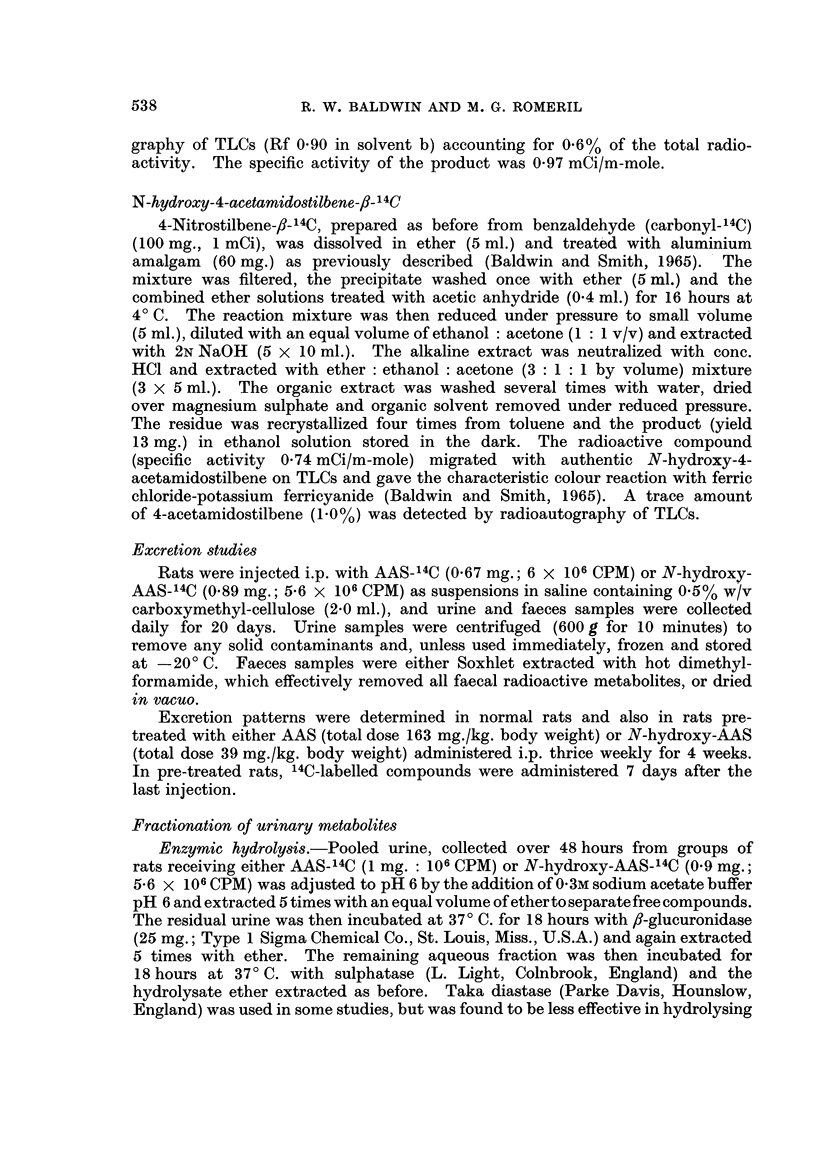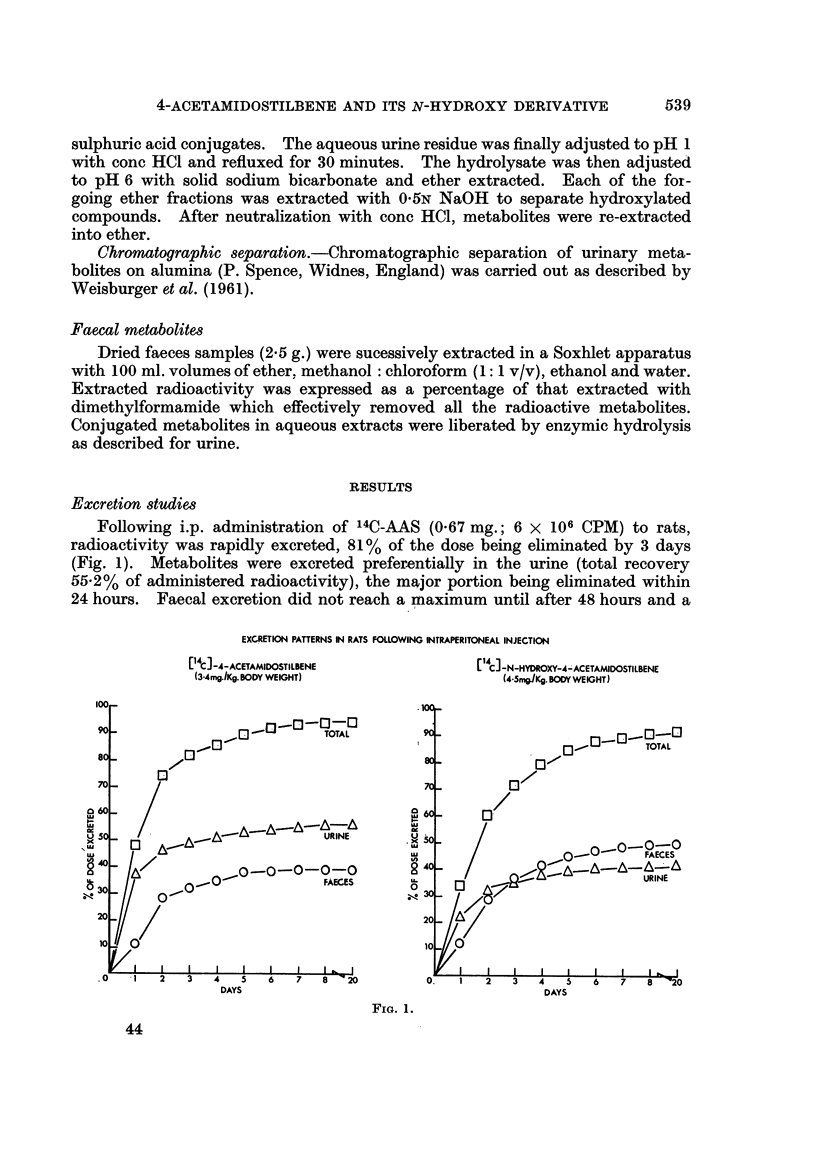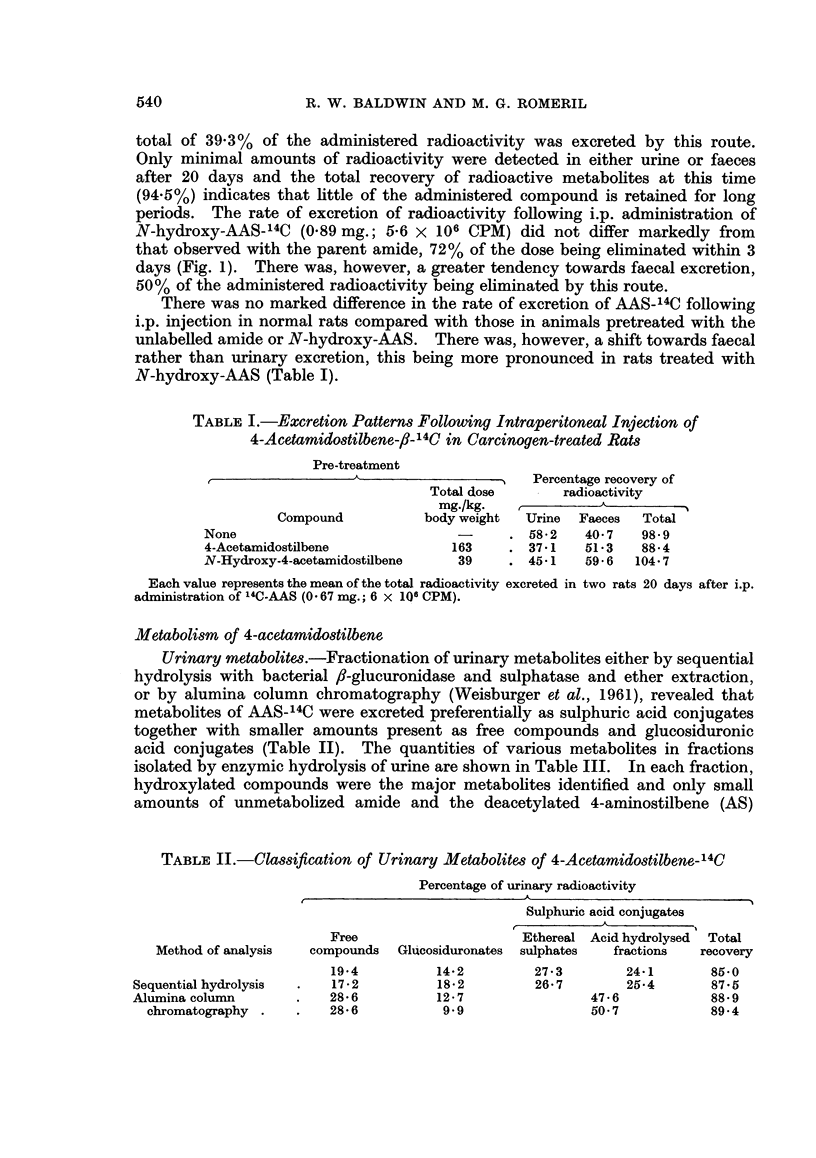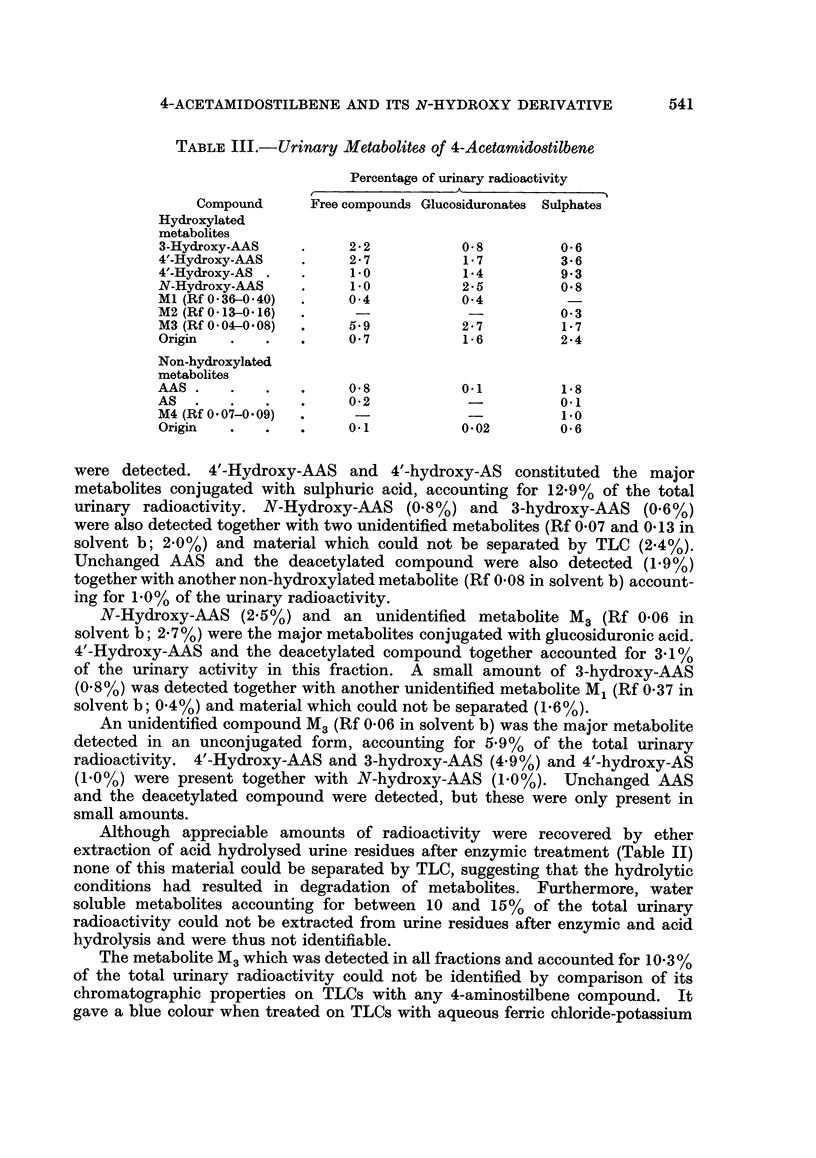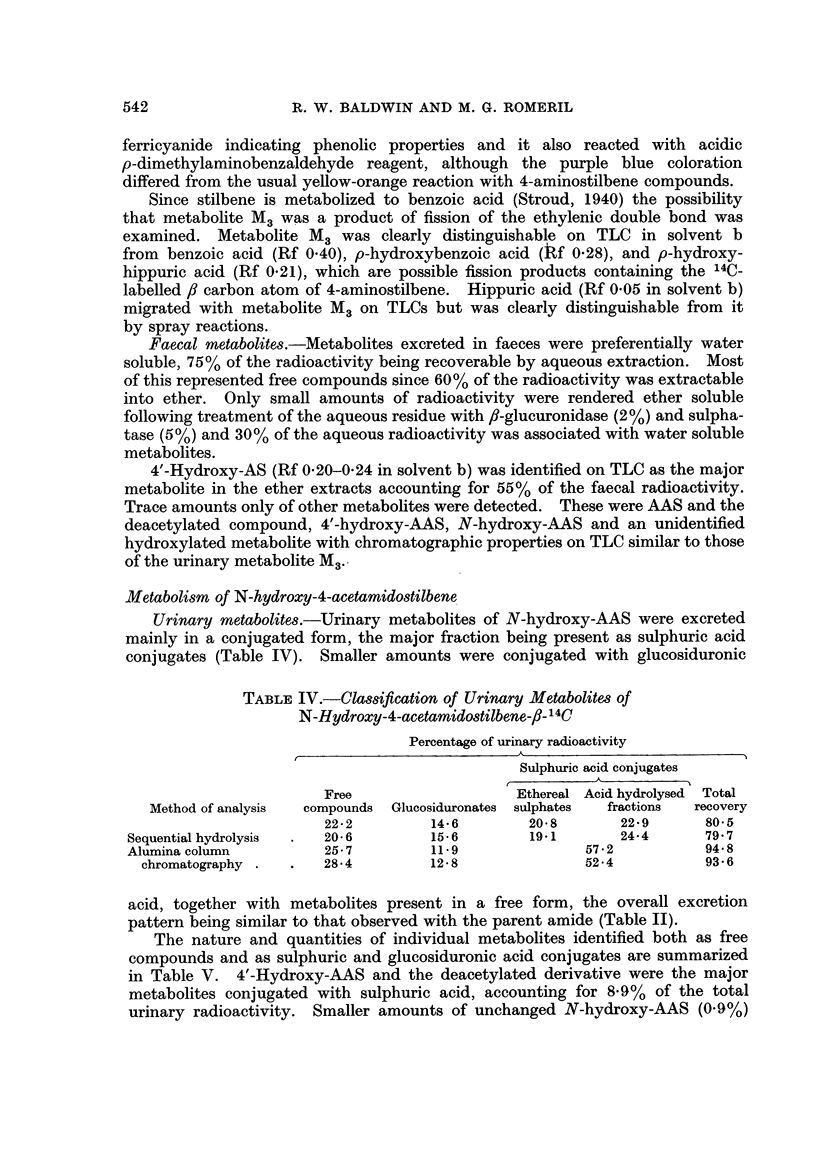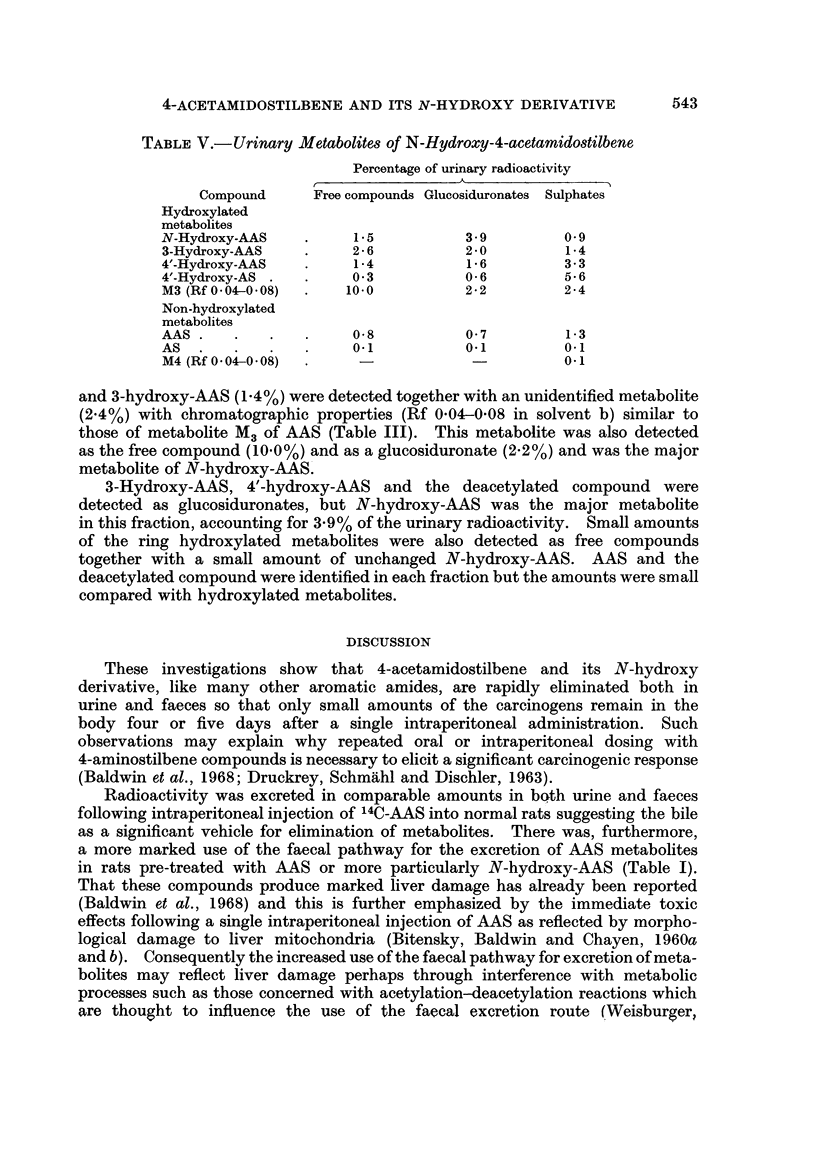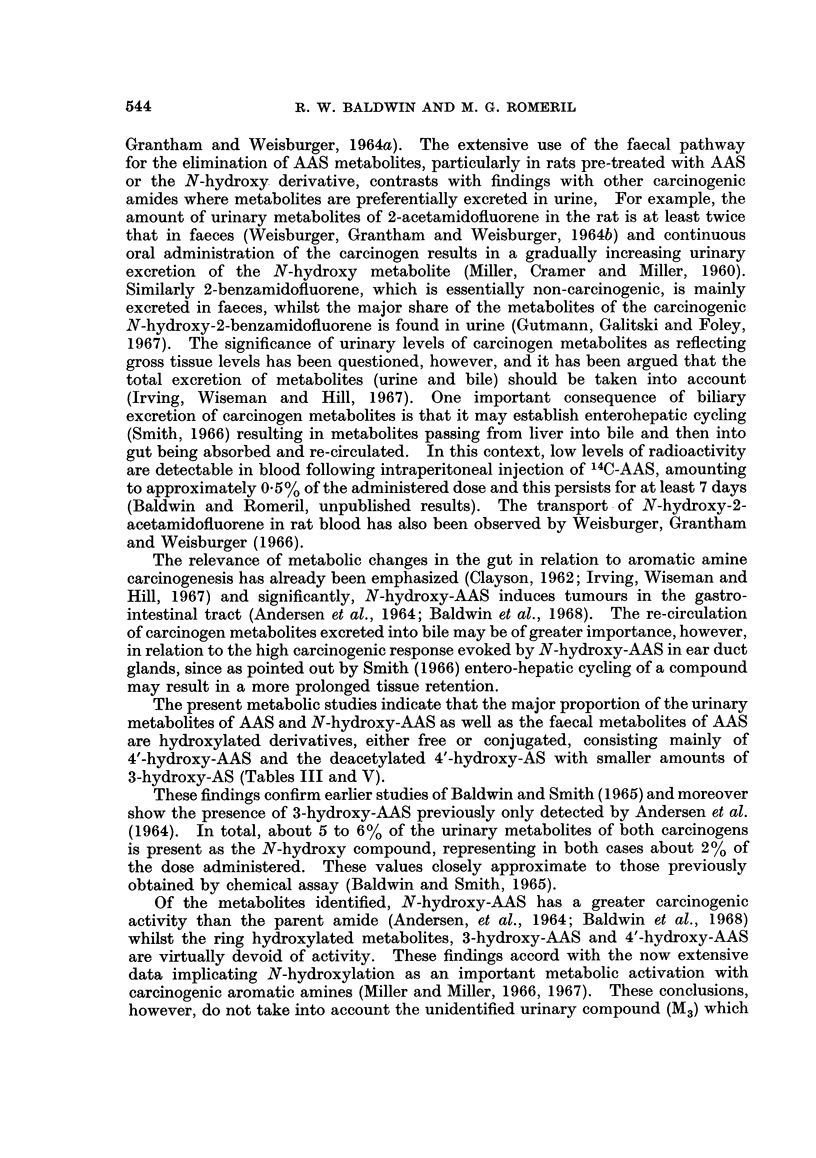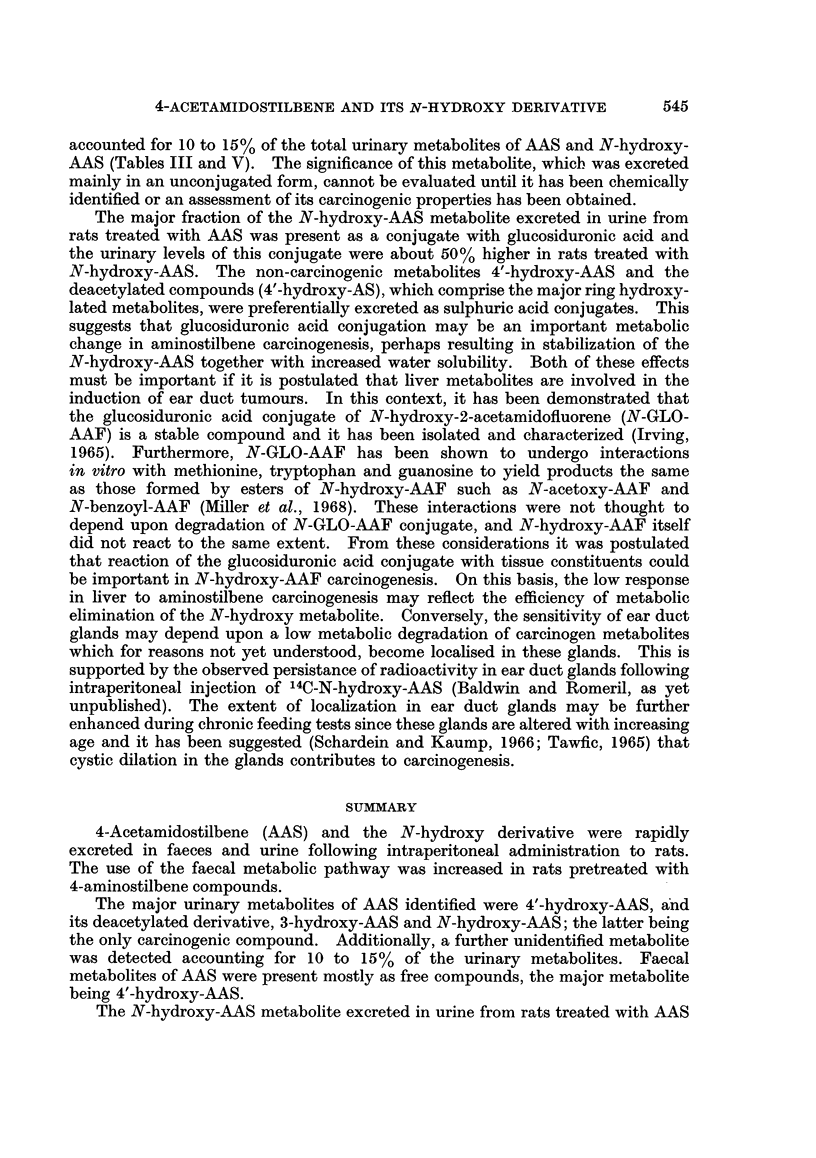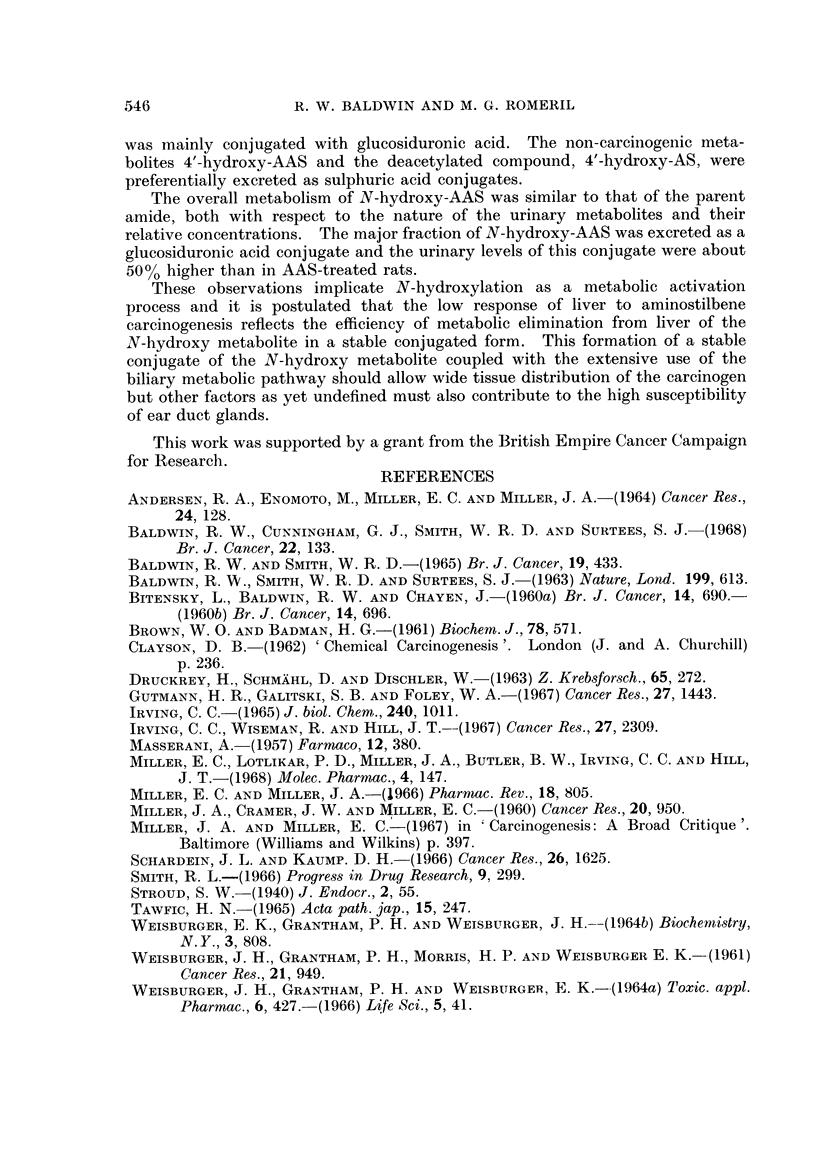# The metabolism of 4-acetamidostilbene and its N-hydroxy derivative.

**DOI:** 10.1038/bjc.1969.66

**Published:** 1969-09

**Authors:** R. W. Baldwin, M. G. Romeril


### THE METABOLISM OF 4-ACETAMIDOSTILBENE AND ITS N-HYDROXY DERIVATIVE

R. W. BALDWIN AND M. G. ROMERIL

*From the British Empire Cancer Campaign Research Laboratory, University of Nottingham*

Received for publication February 18, 1969

*N*-HYDROXYLATION of 4-acetamidostilbene (AAS) in the rat has been demonstrated and this has been postulated as a carcinogen activation process since *N*-hydroxy-AAS is a more potent carcinogen than the parent amide with a wider spectrum of tissue reactivity (Andersen *et al.*, 1964; Baldwin, Smith and Surtees, 1963; Baldwin and Smith, 1965; Baldwin *et al.*, 1968). In contrast, two other hydroxylated metabolites 3-hydroxy-AAS and 4′-hydroxy-AAS are almost completely inactive (Andersen *et al.*, 1964; Baldwin *et al.*, 1968).

Whilst, however, the liver is involved in the metabolic formation of *N*-hydroxylated compounds and both AAS and *N*-hydroxy-AAS produce marked liver damage, the carcinogenic response to these compounds in this tissue is low, tumours generally arising in sebaceous glands associated with the external auditory meatus (Baldwin *et al.*, 1968). If the mechanism of action of 4-aminostilbene compounds is envisaged as involving direct tissue interactions in a manner analogous to that proposed with other classes of chemical carcinogens (Miller and Miller, 1966, 1967) the reactive metabolites must present in these ear duct glands. This may result from direct metabolism of carcinogen in the glands or, more likely, follow liberation of a metabolite formed in the liver and transported as a stable conjugate. In order to examine the latter possibility, the overall metabolism of AAS and the *N*-hydroxy-AAS has been studied in greater detail, particularly with respect to the formation of conjugated metabolites.

These studies have been carried out with $^{14}C$-labelled compounds so that a quantitative assessment of the amounts of individual metabolites has been obtained. Furthermore, the use of $^{14}C$-labelled compounds has permitted the identification of metabolites not detectable by the fluorimetric and colorimetic methods used in earlier studies (Andersen *et al.*, 1964; Baldwin and Smith, 1965).

### MATERIALS AND METHODS

### *Animals*

Adult male rats of an inbred Wistar strain (body weight 160 to 200 g.) were used. These were kept in pairs in all-glass metabolism cages permitting the separate collection of urine and faeces whilst providing free access to food (cubed diet, MRC 41B) and water.

### *Spectra*

Absorption spectra were measured on a Unicam S.P. 500 spectrophotometer and fluorescence spectra on an Aminco Bowman spectrofluorimeter.

*Measurement of radioactivity*

Radioactivity was estimated in a Panax scintillation counter type SC-LP (Panax Equipment Ltd., Redhill, Surrey) at 4° C. The counting efficiency was determined by internal standardization and all counts were corrected to 100% efficiency.

Organic samples (0·1 ml.) were estimated in NE 213 liquid scintillator (4·0 ml.; Nuclear Enterprises Ltd., Edinburgh). Tissue, urine and faeces samples were digested in NKOH and aliquots (0·1 ml.) assayed for radioactivity as described by Brown and Badman (1961).

*Thin layer chromatography* (TLC)

Thin layer chromatograms, prepared from silica gel, were developed with either (a) benzene : ethanol (9 : 1 v/v) or (b) benzene : acetone (3 : 1 v/v). The chromatographic properties of 4-aminostilbene compounds in these solvent systems have been described previously (Baldwin and Smith 1965). Metabolites were detected by their blue fluorescence under ultra-violet light or by spray reactions. Radioactive metabolites were located either by scanning in a Packard scanner, Model 7201 (Packard Instrument Co., Inc., Ill., U.S.A.) or by the darkening of X-ray film (Ilford Industrial G., Ilford Ltd., England) exposed to the plates.

For quantitative estimation, zones of silica gel containing metabolites were removed from the thin layer plates, packed into microcolumns and eluted with ethanol for radioactive assay.

*Preparation of compounds*

Only the *trans* isomers of 4-aminostilbene and its derivaties were studied. 4-acetamidostilbene (AAS) m.p. 234° C. 4′-hydroxy-AAS, m.p. 238° C. and *N*-hydroxy-AAS, m.p. 200° C. were prepared as previously described (Baldwin and Smith, 1965). 4′-Hydroxy-4-aminostilbene (4′-hydroxy-AS) m.p. 273° C. (Masserani 1957) and 3-hydroxy-AAS, m.p. 211–213° C. (Andersen *et al.*, 1964) were prepared by the published methods.

*4-Acetamidostilbene-β-$^{14}C$*

4-Nitrostilbene-β-$^{14}$C was prepared by the condensation of *p*-nitrophenylacetic acid (100 mg.) with benzaldehyde (carbonyl-$^{14}$C) (Radiochemical Centre, Amersham, Bucks., England; 0·5 mCi, 53 mg.) in the presence of piperidine (0·15 ml.) at 140° C. for 2 hours. The mixture was dissolved in ethanol and percolated through a silica gel column (Mallinckrodt, New York, U.S.A., 100 mesh) to separate the blue fluorescent 4-nitrostilbene. The ethanol solution reduced to small volume (5 ml.), was warmed to 65° C., treated with hydrazine hydrate (0·2 ml.) and small portions of freshly prepared Raney nickel added over a one hour period with stirring. The hot solution was immediately centrifuged (600 *g* for 5 minutes) and the supernatant treated with a 10 moles excess of acetic anhydride (0·25 ml.) and refluxed for one hour. The resulting solution was poured into cold water and the precipitated compound collected by filtration, redissolved in ethanol and chromatographed on a silica gel column (yield 59 mg.). The compound migrated with authentic 4-acetamidostilbene on TLCs and had the identical absorption (maximum at 323 mμ in ethanol; E. $3{\cdot}5 \times 10^4$) and fluorescence (maximum at 390 mμ) spectra. A trace impurity was detected by radioautography of TLCs (Rf 0·90 in solvent b) accounting for 0·6% of the total radioactivity. The specific activity of the product was 0·97 mCi/m-mole.

N-*hydroxy-4-acetamidostilbene-β-$^{14}C$*

4-Nitrostilbene-β-$^{14}C$, prepared as before from benzaldehyde (carbonyl-$^{14}C$) (100 mg., 1 mCi), was dissolved in ether (5 ml.) and treated with aluminium amalgam (60 mg.) as previously described (Baldwin and Smith, 1965). The mixture was filtered, the precipitate washed once with ether (5 ml.) and the combined ether solutions treated with acetic anhydride (0·4 ml.) for 16 hours at 4° C. The reaction mixture was then reduced under pressure to small volume (5 ml.), diluted with an equal volume of ethanol : acetone (1 : 1 v/v) and extracted with 2N NaOH (5 × 10 ml.). The alkaline extract was neutralized with conc. HCl and extracted with ether : ethanol : acetone (3 : 1 : 1 by volume) mixture (3 × 5 ml.). The organic extract was washed several times with water, dried over magnesium sulphate and organic solvent removed under reduced pressure. The residue was recrystallized four times from toluene and the product (yield 13 mg.) in ethanol solution stored in the dark. The radioactive compound (specific activity 0·74 mCi/m-mole) migrated with authentic *N*-hydroxy-4-acetamidostilbene on TLCs and gave the characteristic colour reaction with ferric chloride-potassium ferricyanide (Baldwin and Smith, 1965). A trace amount of 4-acetamidostilbene (1·0%) was detected by radioautography of TLCs.

*Excretion studies*

Rats were injected i.p. with AAS-$^{14}C$ (0·67 mg.; 6 × $10^6$ CPM) or *N*-hydroxy-AAS-$^{14}C$ (0·89 mg.; 5·6 × $10^6$ CPM) as suspensions in saline containing 0·5% w/v carboxymethyl-cellulose (2·0 ml.), and urine and faeces samples were collected daily for 20 days. Urine samples were centrifuged (600 *g* for 10 minutes) to remove any solid contaminants and, unless used immediately, frozen and stored at −20° C. Faeces samples were either Soxhlet extracted with hot dimethylformamide, which effectively removed all faecal radioactive metabolites, or dried *in vacuo*.

Excretion patterns were determined in normal rats and also in rats pretreated with either AAS (total dose 163 mg./kg. body weight) or *N*-hydroxy-AAS (total dose 39 mg./kg. body weight) administered i.p. thrice weekly for 4 weeks. In pre-treated rats, $^{14}C$-labelled compounds were administered 7 days after the last injection.

*Fractionation of urinary metabolites*

*Enzymic hydrolysis*.—Pooled urine, collected over 48 hours from groups of rats receiving either AAS-$^{14}C$ (1 mg. : $10^6$ CPM) or *N*-hydroxy-AAS-$^{14}C$ (0·9 mg.; 5·6 × $10^6$ CPM) was adjusted to pH 6 by the addition of 0·3M sodium acetate buffer pH 6 and extracted 5 times with an equal volume of ether to separate free compounds. The residual urine was then incubated at 37° C. for 18 hours with β-glucuronidase (25 mg.; Type 1 Sigma Chemical Co., St. Louis, Miss., U.S.A.) and again extracted 5 times with ether. The remaining aqueous fraction was then incubated for 18 hours at 37° C. with sulphatase (L. Light, Colnbrook, England) and the hydrolysate ether extracted as before. Taka diastase (Parke Davis, Hounslow, England) was used in some studies, but was found to be less effective in hydrolysing sulphuric acid conjugates. The aqueous urine residue was finally adjusted to pH 1 with conc HCl and refluxed for 30 minutes. The hydrolysate was then adjusted to pH 6 with solid sodium bicarbonate and ether extracted. Each of the forgoing ether fractions was extracted with 0·5N NaOH to separate hydroxylated compounds. After neutralization with conc HCl, metabolites were re-extracted into ether.

*Chromatographic separation.*—Chromatographic separation of urinary metabolites on alumina (P. Spence, Widnes, England) was carried out as described by Weisburger *et al.* (1961).

### *Faecal metabolites*

Dried faeces samples (2·5 g.) were sucessively extracted in a Soxhlet apparatus with 100 ml. volumes of ether, methanol : chloroform (1 : 1 v/v), ethanol and water. Extracted radioactivity was expressed as a percentage of that extracted with dimethylformamide which effectively removed all the radioactive metabolites. Conjugated metabolites in aqueous extracts were liberated by enzymic hydrolysis as described for urine.

### RESULTS

### *Excretion studies*

Following i.p. administration of $^{14}$C-AAS (0·67 mg.; 6 $\times$ $10^6$ CPM) to rats, radioactivity was rapidly excreted, 81% of the dose being eliminated by 3 days (Fig. 1). Metabolites were excreted preferentially in the urine (total recovery 55·2% of administered radioactivity), the major portion being eliminated within 24 hours. Faecal excretion did not reach a maximum until after 48 hours and a

Fig. 1.

total of 39·3% of the administered radioactivity was excreted by this route. Only minimal amounts of radioactivity were detected in either urine or faeces after 20 days and the total recovery of radioactive metabolites at this time (94·5%) indicates that little of the administered compound is retained for long periods. The rate of excretion of radioactivity following i.p. administration of *N*-hydroxy-AAS-$^{14}C$ (0·89 mg.; 5·6 × $10^6$ CPM) did not differ markedly from that observed with the parent amide, 72% of the dose being eliminated within 3 days (Fig. 1). There was, however, a greater tendency towards faecal excretion, 50% of the administered radioactivity being eliminated by this route.

There was no marked difference in the rate of excretion of AAS-$^{14}C$ following i.p. injection in normal rats compared with those in animals pretreated with the unlabelled amide or *N*-hydroxy-AAS. There was, however, a shift towards faecal rather than urinary excretion, this being more pronounced in rats treated with *N*-hydroxy-AAS (Table I).

TABLE I.—*Excretion Patterns Following Intraperitoneal Injection of 4-Acetamidostilbene-β-$^{14}C$ in Carcinogen-treated Rats*

| Pre-treatment | | Percentage recovery of radioactivity | | |
|---|---|---|---|---|
| Compound | Total dose mg./kg. body weight | Urine | Faeces | Total |
| None | — | 58·2 | 40·7 | 98·9 |
| 4-Acetamidostilbene | 163 | 37·1 | 51·3 | 88·4 |
| *N*-Hydroxy-4-acetamidostilbene | 39 | 45·1 | 59·6 | 104·7 |

Each value represents the mean of the total radioactivity excreted in two rats 20 days after i.p. administration of $^{14}C$-AAS (0·67 mg.; 6 × $10^6$ CPM).

*Metabolism of 4-acetamidostilbene*

*Urinary metabolites.*—Fractionation of urinary metabolites either by sequential hydrolysis with bacterial β-glucuronidase and sulphatase and ether extraction, or by alumina column chromatography (Weisburger *et al.*, 1961), revealed that metabolites of AAS-$^{14}C$ were excreted preferentially as sulphuric acid conjugates together with smaller amounts present as free compounds and glucosiduronic acid conjugates (Table II). The quantities of various metabolites in fractions isolated by enzymic hydrolysis of urine are shown in Table III. In each fraction, hydroxylated compounds were the major metabolites identified and only small amounts of unmetabolized amide and the deacetylated 4-aminostilbene (AS)

TABLE II.—*Classification of Urinary Metabolites of 4-Acetamidostilbene-$^{14}C$*

| | Percentage of urinary radioactivity | | | | |
|---|---|---|---|---|---|
| | | | Sulphuric acid conjugates | | |
| Method of analysis | Free compounds | Glucosiduronates | Ethereal sulphates | Acid hydrolysed fractions | Total recovery |
| | 19·4 | 14·2 | 27·3 | 24·1 | 85·0 |
| Sequential hydrolysis | 17·2 | 18·2 | 26·7 | 25·4 | 87·5 |
| Alumina column | 28·6 | 12·7 | 47·6 | | 88·9 |
| chromatography | 28·6 | 9·9 | 50·7 | | 89·4 |

TABLE III.—*Urinary Metabolites of 4-Acetamidostilbene*

| Compound | Percentage of urinary radioactivity | | |
| --- | --- | --- | --- |
| | Free compounds | Glucosiduronates | Sulphates |
| Hydroxylated metabolites | | | |
| 3-Hydroxy-AAS | 2·2 | 0·8 | 0·6 |
| 4′-Hydroxy-AAS | 2·7 | 1·7 | 3·6 |
| 4′-Hydroxy-AS | 1·0 | 1·4 | 9·3 |
| *N*-Hydroxy-AAS | 1·0 | 2·5 | 0·8 |
| M1 (Rf 0·36–0·40) | 0·4 | 0·4 | — |
| M2 (Rf 0·13–0·16) | — | — | 0·3 |
| M3 (Rf 0·04–0·08) | 5·9 | 2·7 | 1·7 |
| Origin | 0·7 | 1·6 | 2·4 |
| Non-hydroxylated metabolites | | | |
| AAS | 0·8 | 0·1 | 1·8 |
| AS | 0·2 | — | 0·1 |
| M4 (Rf 0·07–0·09) | — | — | 1·0 |
| Origin | 0·1 | 0·02 | 0·6 |

were detected. 4′-Hydroxy-AAS and 4′-hydroxy-AS constituted the major metabolites conjugated with sulphuric acid, accounting for 12·9% of the total urinary radioactivity. *N*-Hydroxy-AAS (0·8%) and 3-hydroxy-AAS (0·6%) were also detected together with two unidentified metabolites (Rf 0·07 and 0·13 in solvent b; 2·0%) and material which could not be separated by TLC (2·4%). Unchanged AAS and the deacetylated compound were also detected (1·9%) together with another non-hydroxylated metabolite (Rf 0·08 in solvent b) accounting for 1·0% of the urinary radioactivity.

*N*-Hydroxy-AAS (2·5%) and an unidentified metabolite $M_3$ (Rf 0·06 in solvent b; 2·7%) were the major metabolites conjugated with glucosiduronic acid. 4′-Hydroxy-AAS and the deacetylated compound together accounted for 3·1% of the urinary activity in this fraction. A small amount of 3-hydroxy-AAS (0·8%) was detected together with another unidentified metabolite $M_1$ (Rf 0·37 in solvent b; 0·4%) and material which could not be separated (1·6%).

An unidentified compound $M_3$ (Rf 0·06 in solvent b) was the major metabolite detected in an unconjugated form, accounting for 5·9% of the total urinary radioactivity. 4′-Hydroxy-AAS and 3-hydroxy-AAS (4·9%) and 4′-hydroxy-AS (1·0%) were present together with *N*-hydroxy-AAS (1·0%). Unchanged AAS and the deacetylated compound were detected, but these were only present in small amounts.

Although appreciable amounts of radioactivity were recovered by ether extraction of acid hydrolysed urine residues after enzymic treatment (Table II) none of this material could be separated by TLC, suggesting that the hydrolytic conditions had resulted in degradation of metabolites. Furthermore, water soluble metabolites accounting for between 10 and 15% of the total urinary radioactivity could not be extracted from urine residues after enzymic and acid hydrolysis and were thus not identifiable.

The metabolite $M_3$ which was detected in all fractions and accounted for 10·3% of the total urinary radioactivity could not be identified by comparison of its chromatographic properties on TLCs with any 4-aminostilbene compound. It gave a blue colour when treated on TLCs with aqueous ferric chloride-potassium ferricyanide indicating phenolic properties and it also reacted with acidic *p*-dimethylaminobenzaldehyde reagent, although the purple blue coloration differed from the usual yellow-orange reaction with 4-aminostilbene compounds.

Since stilbene is metabolized to benzoic acid (Stroud, 1940) the possibility that metabolite $M_3$ was a product of fission of the ethylenic double bond was examined. Metabolite $M_3$ was clearly distinguishable on TLC in solvent b from benzoic acid (Rf 0·40), *p*-hydroxybenzoic acid (Rf 0·28), and *p*-hydroxyhippuric acid (Rf 0·21), which are possible fission products containing the $^{14}C$-labelled $\beta$ carbon atom of 4-aminostilbene. Hippuric acid (Rf 0·05 in solvent b) migrated with metabolite $M_3$ on TLCs but was clearly distinguishable from it by spray reactions.

*Faecal metabolites.*—Metabolites excreted in faeces were preferentially water soluble, 75% of the radioactivity being recoverable by aqueous extraction. Most of this represented free compounds since 60% of the radioactivity was extractable into ether. Only small amounts of radioactivity were rendered ether soluble following treatment of the aqueous residue with $\beta$-glucuronidase (2%) and sulphatase (5%) and 30% of the aqueous radioactivity was associated with water soluble metabolites.

4′-Hydroxy-AS (Rf 0·20–0·24 in solvent b) was identified on TLC as the major metabolite in the ether extracts accounting for 55% of the faecal radioactivity. Trace amounts only of other metabolites were detected. These were AAS and the deacetylated compound, 4′-hydroxy-AAS, *N*-hydroxy-AAS and an unidentified hydroxylated metabolite with chromatographic properties on TLC similar to those of the urinary metabolite $M_3$.

### *Metabolism of* N-*hydroxy-4-acetamidostilbene*

*Urinary metabolites.*—Urinary metabolites of *N*-hydroxy-AAS were excreted mainly in a conjugated form, the major fraction being present as sulphuric acid conjugates (Table IV). Smaller amounts were conjugated with glucosiduronic

TABLE IV.—*Classification of Urinary Metabolites of* N-*Hydroxy-4-acetamidostilbene-*$\beta$-$^{14}C$

| | Percentage of urinary radioactivity | | | | |
|---|---|---|---|---|---|
| | | | Sulphuric acid conjugates | | |
| Method of analysis | Free compounds | Glucosiduronates | Ethereal sulphates | Acid hydrolysed fractions | Total recovery |
| | 22·2 | 14·6 | 20·8 | 22·9 | 80·5 |
| Sequential hydrolysis | 20·6 | 15·6 | 19·1 | 24·4 | 79·7 |
| Alumina column | 25·7 | 11·9 | | 57·2 | 94·8 |
| chromatography | 28·4 | 12·8 | | 52·4 | 93·6 |

acid, together with metabolites present in a free form, the overall excretion pattern being similar to that observed with the parent amide (Table II).

The nature and quantities of individual metabolites identified both as free compounds and as sulphuric and glucosiduronic acid conjugates are summarized in Table V. 4′-Hydroxy-AAS and the deacetylated derivative were the major metabolites conjugated with sulphuric acid, accounting for 8·9% of the total urinary radioactivity. Smaller amounts of unchanged *N*-hydroxy-AAS (0·9%)

TABLE V.—*Urinary Metabolites of* N-*Hydroxy*-4-*acetamidostilbene*

| Compound | Percentage of urinary radioactivity | | |
|---|---|---|---|
| | Free compounds | Glucosiduronates | Sulphates |
| Hydroxylated metabolites | | | |
| *N*-Hydroxy-AAS | 1·5 | 3·9 | 0·9 |
| 3-Hydroxy-AAS | 2·6 | 2·0 | 1·4 |
| 4′-Hydroxy-AAS | 1·4 | 1·6 | 3·3 |
| 4′-Hydroxy-AS | 0·3 | 0·6 | 5·6 |
| M3 (Rf 0·04–0·08) | 10·0 | 2·2 | 2·4 |
| Non-hydroxylated metabolites | | | |
| AAS | 0·8 | 0·7 | 1·3 |
| AS | 0·1 | 0·1 | 0·1 |
| M4 (Rf 0·04–0·08) | — | — | 0·1 |

and 3-hydroxy-AAS (1·4%) were detected together with an unidentified metabolite (2·4%) with chromatographic properties (Rf 0·04–0·08 in solvent b) similar to those of metabolite $M_3$ of AAS (Table III). This metabolite was also detected as the free compound (10·0%) and as a glucosiduronate (2·2%) and was the major metabolite of *N*-hydroxy-AAS.

3-Hydroxy-AAS, 4′-hydroxy-AAS and the deacetylated compound were detected as glucosiduronates, but *N*-hydroxy-AAS was the major metabolite in this fraction, accounting for 3·9% of the urinary radioactivity. Small amounts of the ring hydroxylated metabolites were also detected as free compounds together with a small amount of unchanged *N*-hydroxy-AAS. AAS and the deacetylated compound were identified in each fraction but the amounts were small compared with hydroxylated metabolites.

### DISCUSSION

These investigations show that 4-acetamidostilbene and its *N*-hydroxy derivative, like many other aromatic amides, are rapidly eliminated both in urine and faeces so that only small amounts of the carcinogens remain in the body four or five days after a single intraperitoneal administration. Such observations may explain why repeated oral or intraperitoneal dosing with 4-aminostilbene compounds is necessary to elicit a significant carcinogenic response (Baldwin *et al.*, 1968; Druckrey, Schmähl and Dischler, 1963).

Radioactivity was excreted in comparable amounts in both urine and faeces following intraperitoneal injection of $^{14}$C-AAS into normal rats suggesting the bile as a significant vehicle for elimination of metabolites. There was, furthermore, a more marked use of the faecal pathway for the excretion of AAS metabolites in rats pre-treated with AAS or more particularly *N*-hydroxy-AAS (Table I). That these compounds produce marked liver damage has already been reported (Baldwin *et al.*, 1968) and this is further emphasized by the immediate toxic effects following a single intraperitoneal injection of AAS as reflected by morphological damage to liver mitochondria (Bitensky, Baldwin and Chayen, 1960*a* and *b*). Consequently the increased use of the faecal pathway for excretion of metabolites may reflect liver damage perhaps through interference with metabolic processes such as those concerned with acetylation–deacetylation reactions which are thought to influence the use of the faecal excretion route (Weisburger,

Grantham and Weisburger, 1964*a*). The extensive use of the faecal pathway for the elimination of AAS metabolites, particularly in rats pre-treated with AAS or the *N*-hydroxy derivative, contrasts with findings with other carcinogenic amides where metabolites are preferentially excreted in urine, For example, the amount of urinary metabolites of 2-acetamidofluorene in the rat is at least twice that in faeces (Weisburger, Grantham and Weisburger, 1964*b*) and continuous oral administration of the carcinogen results in a gradually increasing urinary excretion of the *N*-hydroxy metabolite (Miller, Cramer and Miller, 1960). Similarly 2-benzamidofluorene, which is essentially non-carcinogenic, is mainly excreted in faeces, whilst the major share of the metabolites of the carcinogenic *N*-hydroxy-2-benzamidofluorene is found in urine (Gutmann, Galitski and Foley, 1967). The significance of urinary levels of carcinogen metabolites as reflecting gross tissue levels has been questioned, however, and it has been argued that the total excretion of metabolites (urine and bile) should be taken into account (Irving, Wiseman and Hill, 1967). One important consequence of biliary excretion of carcinogen metabolites is that it may establish enterohepatic cycling (Smith, 1966) resulting in metabolites passing from liver into bile and then into gut being absorbed and re-circulated. In this context, low levels of radioactivity are detectable in blood following intraperitoneal injection of $^{14}$C-AAS, amounting to approximately 0·5% of the administered dose and this persists for at least 7 days (Baldwin and Romeril, unpublished results). The transport of *N*-hydroxy-2-acetamidofluorene in rat blood has also been observed by Weisburger, Grantham and Weisburger (1966).

The relevance of metabolic changes in the gut in relation to aromatic amine carcinogenesis has already been emphasized (Clayson, 1962; Irving, Wiseman and Hill, 1967) and significantly, *N*-hydroxy-AAS induces tumours in the gastro-intestinal tract (Andersen *et al.*, 1964; Baldwin *et al.*, 1968). The re-circulation of carcinogen metabolites excreted into bile may be of greater importance, however, in relation to the high carcinogenic response evoked by *N*-hydroxy-AAS in ear duct glands, since as pointed out by Smith (1966) entero-hepatic cycling of a compound may result in a more prolonged tissue retention.

The present metabolic studies indicate that the major proportion of the urinary metabolites of AAS and *N*-hydroxy-AAS as well as the faecal metabolites of AAS are hydroxylated derivatives, either free or conjugated, consisting mainly of 4′-hydroxy-AAS and the deacetylated 4′-hydroxy-AS with smaller amounts of 3-hydroxy-AS (Tables III and V).

These findings confirm earlier studies of Baldwin and Smith (1965) and moreover show the presence of 3-hydroxy-AAS previously only detected by Andersen *et al.* (1964). In total, about 5 to 6% of the urinary metabolites of both carcinogens is present as the *N*-hydroxy compound, representing in both cases about 2% of the dose administered. These values closely approximate to those previously obtained by chemical assay (Baldwin and Smith, 1965).

Of the metabolites identified, *N*-hydroxy-AAS has a greater carcinogenic activity than the parent amide (Andersen, *et al.*, 1964; Baldwin *et al.*, 1968) whilst the ring hydroxylated metabolites, 3-hydroxy-AAS and 4′-hydroxy-AAS are virtually devoid of activity. These findings accord with the now extensive data implicating *N*-hydroxylation as an important metabolic activation with carcinogenic aromatic amines (Miller and Miller, 1966, 1967). These conclusions, however, do not take into account the unidentified urinary compound ($M_3$) which accounted for 10 to 15% of the total urinary metabolites of AAS and *N*-hydroxy-AAS (Tables III and V). The significance of this metabolite, which was excreted mainly in an unconjugated form, cannot be evaluated until it has been chemically identified or an assessment of its carcinogenic properties has been obtained.

The major fraction of the *N*-hydroxy-AAS metabolite excreted in urine from rats treated with AAS was present as a conjugate with glucosiduronic acid and the urinary levels of this conjugate were about 50% higher in rats treated with *N*-hydroxy-AAS. The non-carcinogenic metabolites 4′-hydroxy-AAS and the deacetylated compounds (4′-hydroxy-AS), which comprise the major ring hydroxylated metabolites, were preferentially excreted as sulphuric acid conjugates. This suggests that glucosiduronic acid conjugation may be an important metabolic change in aminostilbene carcinogenesis, perhaps resulting in stabilization of the *N*-hydroxy-AAS together with increased water solubility. Both of these effects must be important if it is postulated that liver metabolites are involved in the induction of ear duct tumours. In this context, it has been demonstrated that the glucosiduronic acid conjugate of *N*-hydroxy-2-acetamidofluorene (*N*-GLO-AAF) is a stable compound and it has been isolated and characterized (Irving, 1965). Furthermore, *N*-GLO-AAF has been shown to undergo interactions *in vitro* with methionine, tryptophan and guanosine to yield products the same as those formed by esters of *N*-hydroxy-AAF such as *N*-acetoxy-AAF and *N*-benzoyl-AAF (Miller *et al.*, 1968). These interactions were not thought to depend upon degradation of *N*-GLO-AAF conjugate, and *N*-hydroxy-AAF itself did not react to the same extent. From these considerations it was postulated that reaction of the glucosiduronic acid conjugate with tissue constituents could be important in *N*-hydroxy-AAF carcinogenesis. On this basis, the low response in liver to aminostilbene carcinogenesis may reflect the efficiency of metabolic elimination of the *N*-hydroxy metabolite. Conversely, the sensitivity of ear duct glands may depend upon a low metabolic degradation of carcinogen metabolites which for reasons not yet understood, become localised in these glands. This is supported by the observed persistance of radioactivity in ear duct glands following intraperitoneal injection of $^{14}C$-N-hydroxy-AAS (Baldwin and Romeril, as yet unpublished). The extent of localization in ear duct glands may be further enhanced during chronic feeding tests since these glands are altered with increasing age and it has been suggested (Schardein and Kaump, 1966; Tawfic, 1965) that cystic dilation in the glands contributes to carcinogenesis.

### SUMMARY

4-Acetamidostilbene (AAS) and the *N*-hydroxy derivative were rapidly excreted in faeces and urine following intraperitoneal administration to rats. The use of the faecal metabolic pathway was increased in rats pretreated with 4-aminostilbene compounds.

The major urinary metabolites of AAS identified were 4′-hydroxy-AAS, and its deacetylated derivative, 3-hydroxy-AAS and *N*-hydroxy-AAS; the latter being the only carcinogenic compound. Additionally, a further unidentified metabolite was detected accounting for 10 to 15% of the urinary metabolites. Faecal metabolites of AAS were present mostly as free compounds, the major metabolite being 4′-hydroxy-AAS.

The *N*-hydroxy-AAS metabolite excreted in urine from rats treated with AAS

was mainly conjugated with glucosiduronic acid. The non-carcinogenic metabolites 4′-hydroxy-AAS and the deacetylated compound, 4′-hydroxy-AS, were preferentially excreted as sulphuric acid conjugates.

The overall metabolism of *N*-hydroxy-AAS was similar to that of the parent amide, both with respect to the nature of the urinary metabolites and their relative concentrations. The major fraction of *N*-hydroxy-AAS was excreted as a glucosiduronic acid conjugate and the urinary levels of this conjugate were about 50% higher than in AAS-treated rats.

These observations implicate *N*-hydroxylation as a metabolic activation process and it is postulated that the low response of liver to aminostilbene carcinogenesis reflects the efficiency of metabolic elimination from liver of the *N*-hydroxy metabolite in a stable conjugated form. This formation of a stable conjugate of the *N*-hydroxy metabolite coupled with the extensive use of the biliary metabolic pathway should allow wide tissue distribution of the carcinogen but other factors as yet undefined must also contribute to the high susceptibility of ear duct glands.

This work was supported by a grant from the British Empire Cancer Campaign for Research.

### REFERENCES

Andersen, R. A., Enomoto, M., Miller, E. C. and Miller, J. A.—(1964) *Cancer Res.*, **24**, 128.

Baldwin, R. W., Cunningham, G. J., Smith, W. R. D. and Surtees, S. J.—(1968) *Br. J. Cancer*, **22**, 133.

Baldwin, R. W. and Smith, W. R. D.—(1965) *Br. J. Cancer*, **19**, 433.

Baldwin, R. W., Smith, W. R. D. and Surtees, S. J.—(1963) *Nature, Lond.* **199**, 613.

Bitensky, L., Baldwin, R. W. and Chayen, J.—(1960*a*) *Br. J. Cancer*, **14**, 690.—(1960*b*) *Br. J. Cancer*, **14**, 696.

Brown, W. O. and Badman, H. G.—(1961) *Biochem. J.*, **78**, 571.

Clayson, D. B.—(1962) 'Chemical Carcinogenesis'. London (J. and A. Churchill) p. 236.

Druckrey, H., Schmähl, D. and Dischler, W.—(1963) *Z. Krebsforsch.*, **65**, 272.

Gutmann, H. R., Galitski, S. B. and Foley, W. A.—(1967) *Cancer Res.*, **27**, 1443.

Irving, C. C.—(1965) *J. biol. Chem.*, **240**, 1011.

Irving, C. C., Wiseman, R. and Hill, J. T.—(1967) *Cancer Res.*, **27**, 2309.

Masserani, A.—(1957) *Farmaco*, **12**, 380.

Miller, E. C., Lotlikar, P. D., Miller, J. A., Butler, B. W., Irving, C. C. and Hill, J. T.—(1968) *Molec. Pharmac.*, **4**, 147.

Miller, E. C. and Miller, J. A.—(1966) *Pharmac. Rev.*, **18**, 805.

Miller, J. A., Cramer, J. W. and Miller, E. C.—(1960) *Cancer Res.*, **20**, 950.

Miller, J. A. and Miller, E. C.—(1967) in 'Carcinogenesis: A Broad Critique'. Baltimore (Williams and Wilkins) p. 397.

Schardein, J. L. and Kaump. D. H.—(1966) *Cancer Res.*, **26**, 1625.

Smith, R. L.—(1966) *Progress in Drug Research*, **9**, 299.

Stroud, S. W.—(1940) *J. Endocr.*, **2**, 55.

Tawfic, H. N.—(1965) *Acta path. jap.*, **15**, 247.

Weisburger, E. K., Grantham, P. H. and Weisburger, J. H.—(1964*b*) *Biochemistry, N.Y.*, **3**, 808.

Weisburger, J. H., Grantham, P. H., Morris, H. P. and Weisburger E. K.—(1961) *Cancer Res.*, **21**, 949.

Weisburger, J. H., Grantham, P. H. and Weisburger, E. K.—(1964*a*) *Toxic. appl. Pharmac.*, **6**, 427.—(1966) *Life Sci.*, **5**, 41.